# The Risk of Regurgitation and Pulmonary Aspiration in a Patient after Gastric Banding

**DOI:** 10.1155/2012/186104

**Published:** 2012-03-26

**Authors:** Abdulvahab Thekkethodika

**Affiliations:** Department of Anesthesia ICU, Pain Management and Palliative Care, Hamad Medical Corporation, 3050 Doha, Qatar

## Abstract

Laparoscopic gastric banding is a popular surgical treatment performed to control morbid obesity all over the world. Regurgitation of food material from stomach is very common in these patients. Remnants of food material may risk the airway for pulmonary aspiration. This case experience shows that despite the extended fasting period, airway is not protected from the risk of aspiration. Delayed gastric emptying and altered gastroesophageal motility keep the food materials in the stomach and precipitate regurgitation. So any such patient should be considered as full stomach. Airway manipulation in these patients should be under direct laryngoscopic vision and rapid sequence induction with endotracheal intubation should be considered as mandatory for general anesthesia.

## 1. Introduction

Obesity is a growing health problem throughout the world. It is more prevalent in the developed countries. Morbid obesity is defined as body mass index (BMI) 40 or more [1]. It has a lot of medical and anesthetic implications. To control obesity, dietary restrictions are practiced. Most of the time, it does not work. 

Laparoscopic gastric banding is a surgical method of treating obesity in patients unable to lose weight by diet control. A ring is put over the cardia of stomach to limit food intake. This will alter the normal physiology of gastroesophageal junction. Impaired gastroesophageal peristalsis and delayed gastric emptying may increase the risk of esophageal regurgitation and pulmonary aspiration. This is of serious concern when the patient comes for another surgery requiring general anesthesia. 

We are discussing a case of regurgitation in a gastric-banded patient.

## 2. Case Report

A 33-year-old lady presented for extraction of impacted third molar teeth under general anesthesia 3 years after gastric banding. After having lost 43 kg, she weighed 82 kg for 159 cm height (BMI-32.5). She had no other significant comorbidities and no complaints of altered esophago gastric peristalsis. She had undergone bilateral mammoplasty under general anesthesia using Laryngeal Mask Airway (LMA) 2 months ago. She took her last meal the day before surgery at 2 PM which consisted of rice and chicken meat, and she had taken fruit juice at 10 PM. She was posted as day care surgery. Anesthesia was induced at 9.30 AM, with IV fentanyl 100 mcg, atracurium 35 mg, propofol 150 mg, and ventilated with oxygen and sevoflurane without any difficulty. Ventilation was uneventful and smooth. Direct laryngoscopy was done. Larynx was dry, but there were 3 pieces of undigested 0.5–1.5 cm sized chicken meat seen at the laryngeal inlet. These were removed by Magill's forceps and broken pieces were suctioned out. Later trachea was intubated. Anesthesia and surgery went on uneventfully. The recovery was smooth. In the postanesthesia care unit (PACU), while she was fully awake, she vomited undigested food particles. She was given IV ondansetrone 4 mg. She had no respiratory symptoms in PACU or in the day-care unit. Her chest was clear on examination. She was discharged home after 3 hours from the hospital. 

## 3. Discussion

Normal functioning of lower esophageal sphincter and preserved gastroesophageal peristalsis prevent the gastroesophageal reflux and regurgitation. Several studies have suggested that esophageogastric peristalsis is altered after gastric banding [2–4]. Lower esophageal sphincter malfunction and lower esophageal dilatation also are reported. These physiological modifications will make the patient prone for regurgitation and pulmonary aspiration. Cases of regurgitation during anesthesia and consequent pulmonary aspiration are reported before [5, 6]. Significant weight loss after gastric banding indicates the efficacy of the surgery and such patients have significant physiological alteration.

There are no specific fasting guidelines on anesthetic management for postgastric banding patients. Extended fasting period is widely practiced. In this case she was anesthetized, 19 hours after a solid meal and 11 hours after a liquid meal. Even then the undigested food material was present in the stomach. Increased tendency for regurgitation may be due to abnormal gastroesophageal motility. Beneficiary effect of prokinetic agents and H2 antagonists are to be studied.

Our case experience confirms the possibility of regurgitation in these patients. Regurgitation was not noticed at the time of induction of anesthesia. It might have happened any time after the last meal before anesthesia was induced, might be even during the sleep. Such episodes of regurgitation leave food materials in the upper airway. Mask ventilation or usage of LMA could push the food particles down to the lower airway and the result is disastrous. We suggest a minimum fasting of 24 hours for solid meal and 12 hours for liquids. They may have to be admitted preoperatively to give IV fluids. Prokinetic agents should be given as premedication 12 hour and 1 hour before surgery. Any airway manipulation should be under direct visualization, and rapid sequence induction with endotracheal intubation should be considered mandatory for general anesthesia.

## References

[1] Haslam DW, James WPT (2005). Obesity. *The Lancet*.

[2] Weiss HG, Nehoda H, Labeck B (2000). Treatment of morbid obesity with laparoscopic adjustable gastric banding affects esophageal motility. *American Journal of Surgery*.

[3] Weiss HG, Nehoda H, Labeck B (2002). Adjustable gastric and esophagogastric banding: a randomized clinical trial. *Obesity Surgery*.

[4] Korenkov M, Köhler L, Yücel N (2002). Esophageal motility and reflux symptoms before and after bariatric surgery. *Obesity Surgery*.

[5] Kocian R, Spahn DR (2005). Bronchial aspiration in patients after weight loss due to gastric banding. *Anesthesia and Analgesia*.

[6] Jean J, Compère V, Fourdrinier V (2008). The risk of pulmonary aspiration in patients after weight loss due to bariatric surgery. *Anesthesia and Analgesia*.

